# A Versatile Microencapsulation Platform for Hyaluronic Acid and Polyethylene Glycol

**DOI:** 10.1089/ten.tea.2019.0286

**Published:** 2021-02-15

**Authors:** Stephen Harrington, Lindsey Ott, Francis Karanu, Karthik Ramachandran, Lisa Stehno-Bittel

**Affiliations:** ^1^ Likarda LLC, Kansas City, Missouri, USA.; ^2^ Department of Rehabilitation Science, University of Kansas Medical Center, Kansas City, Kansas, USA.

**Keywords:** microencapsulation, cell therapy, hydrogel, encapsulation, islet transplantation

## Abstract

Cell microencapsulation is a rapidly expanding field with broad potential for stem cell therapies and tissue engineering research. Traditional alginate microspheres suffer from poor biocompatibility, and microencapsulation of more advanced hydrogels is challenging due to their slower gelation rates. We have developed a novel, noncytotoxic, nonemulsion-based method to produce hydrogel microspheres compatible with a wide variety of materials, called core-shell spherification (CSS). Fabrication of microspheres by CSS derived from two slow-hardening hydrogels, hyaluronic acid (HA) and polyethylene glycol diacrylate (PEGDA), was characterized. HA microspheres were manufactured with two different crosslinking methods: thiolation and methacrylation. Microspheres of methacrylated HA (MeHA) had the greatest swelling ratio, the largest average diameter, and the lowest diffusion barrier. In contrast, PEGDA microspheres had the smallest diameters, the lowest swelling ratio, and the highest diffusion barrier, while microspheres of thiolated HA had characteristics that were in between the other two groups. To test the ability of the hydrogels to protect cells, while promoting function, diabetic NOD mice received intraperitoneal injections of PEGDA or MeHA microencapsulated canine islets. PEGDA microspheres reversed diabetes for the length of the study (up to 16 weeks). In contrast, islets encapsulated in MeHA microspheres at the same dose restored normoglycemia, but only transiently (3–4 weeks). Nonencapsulated canine islet transplanted at the same dose did not restore normoglycemia for any length of time. In conclusion, CSS provides a nontoxic microencapsulation procedure compatible with various hydrogel types.

**Impact statement:**

Core-shell spherification, described here for the first time, is a versatile method of coating cells to protect them following transplantation. Before the invention of this technique, only instantaneously hardening hydrogels, like alginate, could be used to encapsulate cells. With this new technology, biocompatible hydrogels can now be used for encapsulation without harsh emulsion chemicals. The technique involves a temporary shell of alginate around the slow-hardening gel that provides time for crosslinking to occur. Subsequently, the alginate shell is easily removed, leaving the cells in a protective microsphere with a higher surface area for diffusion than large encapsulating devices.

## Introduction

The concept of cell encapsulation was introduced in 1980 by Lim and Sun, who showed that islets embedded in alginate microspheres could reverse diabetes in rats without the need for immunosuppression, although for only a few weeks.^1^ Encapsulation with alginate has persisted as the clear material of choice for cell therapies due to alginate's unique, nearly instantaneous, crosslinking kinetics, enabling straightforward fabrication by dropping aqueous sodium alginate into a bath of crosslinking calcium.^2^

However, alginate does present some challenges that researchers have attempted to resolve with numerous variations in the alginate fabrication process. For example, the weakness of the alginate microspheres led researchers to alter the formulation for improved mechanical resistance required during injections.^3^ Others have focused on the poor long-term biocompatibility of calcium alginate,^4–6^ noting that the purification of alginate is essential to its biocompatibility.^7–9^

In contrast, there is an abundance of alternative hydrogels that are synthetically manufactured, eliminating the variation associated with alginate purification steps.^8^ Unfortunately, these hydrogels have slower gelation times than alginate, and thus are typically prepared as macroscopic structures,^10–12^ which can result in diffusion limitations due to the low surface area.^13^

Protocols have been developed for producing microspheres using alternative hydrogels such as polyethylene glycol (PEG), agarose, chitosan, or hyaluronic acid (HA), but are based on oil-emulsion techniques. Often, the emulsion protocols include harsh nonaqueous solvents that can lead to cytotoxicity issues or poor process control, resulting in highly variable microsphere shapes and sizes.^14–16^ Patterned molds are a way to circumvent the cytotoxicity issues and still create microspheres.^17,18^ However, scaling these methods to the levels needed for transplants can be challenging. An excellent review of the different methods used to create microspheres from PEG or HA, including photolithography, micromolding, and emulsion, can be found in Choe *et al.*^19^

We developed a novel method for producing hydrogel microspheres, termed core-shell spherification (CSS). This method was strategically designed for use with hydrogels with much slower gelation rates compared to alginate, enabling the production of microspheres with a variety of chemical, physical, and bioactive properties. Furthermore, the method does not use oil-emulsion techniques and was developed for standard good manufacturing practice (GMP)-ready equipment and materials to better facilitate accessibility, and scale and regulatory compliance. Compared to utilization of micromolds to encapsulate cells, CSS can scale easily with commercially available instruments. We demonstrate microsphere production by CSS using two popular hydrogels, HA and PEG, compare their properties, and evaluate *in vitro* cytotoxicity. Finally, the functional/therapeutic capacity of cells encapsulated by CSS was evaluated by islet xenotransplantation in diabetic mice.

## Materials and Methods

### Isolation, assessment, and culture of canine islets

Canine islets were isolated from pancreas obtained locally from euthanized donors from local veterinary clinics with consent by owners for organ donation. The procurement and digestion protocol have been published previously in detail.^20^ Animals were scheduled for euthanasia for other health reasons and had no endocrine disorders. Euthanasia was performed by a licensed veterinarian overseeing the care of each animal. Collection of the pancreas after death from animals euthanized for reasons other than organ procurement was determined to be exempt from Institutional animal care and use committee (IACUC) review by the University of Kansas Medical Center IACUC.

Following removal and cleaning of the organ, collagenase digestion followed by density gradient purification was performed, following our described protocol.^20^ Isolated islets were converted to islet equivalents, or “IEQ,” for quantification purposes by dithizone staining according to standard published protocols.^21^ Canine islets were cultured in CMRL 1066 supplemented with 10% fetal bovine serum, 2 mM glutamine, 10 mM nicotinamide, and a 1% antibiotic-antimycotic solution at 37°C and 5% CO_2_.

### Fabrication of methacrylated hyaluronic acid hydrogel microspheres

**FIG. 1.**
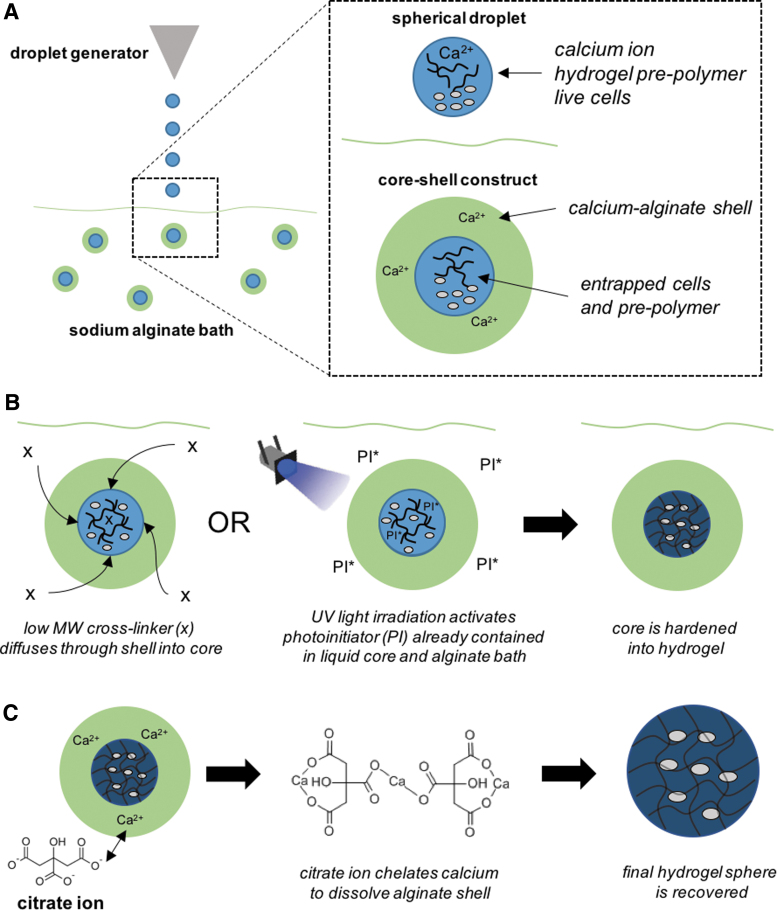
Schematic of core-shell spherification method. **(A)** A hydrogel precursor solution is prepared with calcium chloride and mixed with cells. The precursor is then extruded into an alginate bath using a commercial encapsulator. The calcium in the precursor diffuses into the alginate, resulting in spherical core-shell constructs in which the hydrogel precursor and cells are entrapped within the alginate shell. **(B)** The precursor is crosslinked by diffusion of a small crosslinker through the shell or by activation of a photoinitiator with ultraviolet light. **(C)** In the last step, the alginate shell is dissolved with citrate ion, leaving only the interior crosslinked hydrogel microsphere.

Once nontoxic levels of crosslinkers were determined, microsphere production was initiated. A general schematic of the hydrogel microsphere fabrication process, termed CSS, is provided in Figure 1. The methacrylated HA (MeHA) was manufactured by reacting HA (MW 1 MDa; Lifecore Biomedical) with a 50-fold molar excess glycidyl methacrylate (Sigma) in the presence of triethylamine and tertbutyl ammonium bromide (Sigma) in a 50:50 water:dimethyl sulfoxide mixture for 5 days. MeHA was dialyzed against deionized (DI) water for 2 days and then lyophilized.^23–25^ The degree of methacrylation was determined to be 54–72% using ^1^H NMR (Avance AV-III 500; Bruker) by calculating the ratio of the relative peak area of methacrylate protons to methyl protons.^26^

A low concentration of polyethylene glycol diacrylate (PEGDA) was added to the MeHA to improve crosslinking. The MeHA was mixed with PEGDA (MW 3.4 kDa; Laysan Bio, Inc.), prepared at 2.5%:1% (w/w) in a custom buffer containing 100 mM calcium chloride, 15 mM HEPES, and 0.05% (w/v) Irgacure 2959 with OptiPrep (CosmoBioUSA, Inc.). The solution was passed through a 0.22-μ filter. The viscosity of the precursor solution was measured with a Cannon-Manning Semi-Micro calibrated glass capillary viscometer at room temperature.

The precursor was extruded ∼10 cm by an automated droplet generator into a stirred bath of 0.15% (w/v) sodium alginate (Protanal LF 10/60; FMC Corp.) containing 300 mM mannitol and 0.1% Tween 20, and adjusted to pH 7.6 using a custom 15 mM HEPES buffer. For the MeHA precursor, 0.05% (w/v) Irgacure 2959 was added to the alginate bath. A Buchi 395-Pro Encapsulator (Buchi Corporation, Newcastle, DE) equipped with an air jet nozzle system and a 400-μ diameter inner fluid nozzle within a 1.5 mm concentric air nozzle was used for droplet generation. The droplets were extruded using compressed nitrogen.

The MeHA droplets formed core-shell constructs, which were irradiated with long-wave ultraviolet light to initiate free radical photopolymerization of the MeHA and PEGDA. Irradiance parameters are described above and were set at ∼40 mW/cm^2^ at the center of the bath (PortaRay 400; Uvitron International). Irradiation was applied continuously during extrusion of the precursor solution and for 1 min after extrusion to ensure complete crosslinking of the core-shell constructs. The average width of the alginate shell was 302.9 ± 6.2 μm. Core-shell constructs were next rinsed in a 25 mM citrate buffer in Dulbecco's phosphate-buffered saline (DPBS) for 5 min under mild stirring to dissolve the alginate shells. The resulting microspheres were collected using a steel mesh screen and suspended in DPBS. A second rinse in 50 mM citrate for 5 min was performed to ensure complete dissolution and removal of the alginate shell.

Removal of the sodium alginate shell was confirmed using high-performance liquid chromatography (HPLC) using an Agilent 1100 HPLC system equipped with a multiple wavelength detector. The mobile phase consisted of 40 mM anhydrous dibasic sodium phosphate with the pH of the solution adjusted to 6.0 with NaOH, while the column used was an Agilent ZORBAX Eclipse Plus C18 (5 μm, 150 × 4.6 mm I.D.) column. The detection was carried out at a wavelength of 210 nm with a flow rate of 0.7 mL/min. The injection volume of the standards and samples solution was set at 20 μL.

### Fabrication of thiolated HA hydrogel microspheres

The thiolated HA (ThHA) precursor was prepared by dissolving the ThHA (HyStem; Biotime, Inc.) at 1.2% (w/w) in a custom buffer containing 100 mM calcium chloride and OptiPrep to adjust solution density (CosmoBioUSA) with a pH of 7.0 (adjusted with 15 mM HEPES). The solution viscosity was measured as described above. Core-shell constructs were produced as described above. However, crosslinking was achieved by chemical crosslinking, versus the free radical photopolymerization with MeHA microspheres. Briefly, the precursor solution formed core-shell constructs upon contact with the alginate bath, containing 0.4% PEGDA (MW 3.4 kDa; Laysan Bio, Inc.), which was stirred gently for 5 min.

Crosslinking proceeded by diffusion of the smaller 3.4 kDa PEGDA molecule into the core, through the alginate shell. The bath was then diluted by half with DPBS, which reduced the solution pH to 7.4. Stirring continued for an additional 30 min to crosslink the HA precursor within the core. The microspheres were collected, and the alginate shells were removed as described above.

### Fabrication of polyethylene glycol diacrylate hydrogel microspheres

PEGDA hydrogel precursor solution was prepared by dissolving PEGDA 3400 and 20,000 (Laysan Bio, Inc.) at 18% and 12% (w/w), respectively, in a buffer containing 100 mM calcium chloride, 10 mM HEPES, and 0.025% (w/v) Irgacure 2959. The solution was filtered using a 0.22-μ syringe and the viscosity was measured as described above. The precursor was extruded into a stirred alginate bath as described for the MeHA microspheres, but with 0.025% (w/v) Irgacure 2959. All solutions for MeHA and PEGDA microsphere fabrication were prepared in water degassed by sonication to eliminate excess oxygen, a known photo-crosslinking inhibitor.^27^ Core-shell constructs were irradiated and processed as described above.

### Physical properties and size distribution of hydrogel microspheres

Images of individual microspheres were captured using a Biotek Cytation 5 Multi-mode imaging reader. The microspheres were imaged in multiwell plates in phosphate-buffered saline (PBS), and the images were analyzed in Adobe Photoshop to determine the average microsphere diameter and size distribution for each microsphere type (*N* = 100 microspheres/group). Hydrogel microspheres were further characterized by determination of the swelling ratio, “*Q*,” for each microsphere type, which is the ratio of the swollen hydrated mass to the dry mass.^28^ Microspheres were immersed in excess DI water for 24 h before measurement to remove dissolved salts and to ensure equilibrium swelling was reached. Microspheres were removed of excess surface moisture and then weighed on a preweighed watch glass. Subsequently, the spheres were dried overnight at 60°C and reweighed to obtain the dry mass (*N* = 3/group).

### Diffusion characteristics of microspheres

Hydrogel microspheres were incubated overnight in 0.1 mg/mL solutions of fluorescein isothiocyanate (FITC)-labeled dextrans in DPBS with molecular weights of 10, 40, 70, and 500 kDa (Invitrogen Molecular Probes) following procedures previously published.^29^ Microspheres were rinsed with DPBS and imaged by laser scanning confocal microscopy to monitor efflux of the probes (Olympus Fluoview 300 Laser Scanning Microscope).^22^ Micrographs were captured at set time points between 3 and 150 min after removal of the microspheres from the FITC-dextran incubation solutions.

Analysis of the fluorescence levels was limited to the core of the spheres. The fluorescence intensity within a 100-pixel box at three locations within each bead's core was measured using the histogram feature within Photoshop 2020. Mean pixel intensity within the boxes was recorded and background intensity subtracted. Subsequently, the three measurements were averaged. The rate of fluorescence decline was calculated across the first 30 min of the experiments.

### Encapsulated islet xenotransplants

Immunodeficient NOD/SCID mice (NOD.CB17-*Prkdc^scid^*; Jackson Laboratory) treated with streptozotocin (STZ) to induce diabetes were used to evaluate the functional capacity of canine islets encapsulated by CSS. Mice were rendered diabetic by injection of STZ at 220–250 mg/kg. If hyperglycemia was not reached with a single injection of STZ, a subsequent injection was given. Mice were declared diabetic when nonfasting blood glucose levels reached 350 mg/dL or greater, and were held for a minimum of 5 days after induction of diabetes to allow for clearance of the STZ before receiving islet transplants.

The PEGDA and MeHA formulations of islet-encapsulated microspheres were fabricated as described above with the exception that canine islets were mixed into the precursor. A 1.1 × concentration of the precursor was mixed with a slurry of canine islets at a 10:1 volume ratio just before droplet generation. Fabrication of islet microspheres was done aseptically in a closed, sterile bioreactor system described above.

Before transplantation, encapsulated cells were stained for viability. Microspheres were incubated in calcein AM (4 μM; ThermoFisher) for live cell identification and in propidium iodide (PI) for dead cell identification (1 μg/mL; ThermoFisher) for 30 min. Fluorescence micrographs were captured with a Cytation 5 Imaging Multi-Mode Reader (Biotek Instruments). Using the Cytation software, the area of calcein-stained (live) cells was divided by the total cell area (obtained with bright field images of the same fields) within the microspheres, resulting in the percentage of live cells. Images obtained 1 and 5 days after encapsulation were analyzed.

Six mice received 4000 IEQ of islets encapsulated in PEGDA, and four mice received the same dose of islets encapsulated in MeHA. Two mice were transplanted (intraperitoneal [IP]) with an equivalent dose of unencapsulated islets as controls. The microspheres were administered in a transplant medium consisting of CMRL 1066 without phenol red, with 10 mM nicotinamide and 2 mM l-glutamine (Corning GlutaGro), and injected through a syringe attached to an 18G catheter into the IP space of the mice. The total packed volume of the transplant was ∼3 mL. The islet dose was based on previous escalating-dose studies, demonstrating that ∼4000 IEQ per mouse resulted in normoglycemia, and is consistent with previous publications of canine islets transplanted into mice.^30,31^ Blood glucose levels were measured daily for 14 days following transplant for all mice, and biweekly after that.

At 10 weeks into the study, all the mice receiving MeHA encapsulated islets had returned to hyperglycemia and were terminated, along with the unencapsulated islet group. Four mice from the PEGDA group were also terminated at 10 weeks to serve as comparisons for subsequent histology studies. The two remaining PEGDA mice were followed for another 6 weeks. At the time of euthanasia, microspheres were recovered from the IP space and placed in culture medium for evaluation. Islets within the microspheres were stained with dithizone to detect the presence of insulin,^21^ as well as with calcein (live cells) and PI (apoptotic/necrotic cells) to evaluate cell viability of the explanted tissues. Color, bright field, and fluorescent images of the stained microspheres were captured using a Cytation 5 Imaging Multi-Mode Reader (Biotek Instruments, Inc.).

Tissue samples with attached microbeads were removed and fixed overnight in 10% neutral buffered formalin before transferring to PBS. Tissues were embedded in paraffin blocks and appropriately sectioned for staining. For hematoxylin and eosin (H&E) staining, sections were serially deparaffinized by placing in Clear Rite 3 solutions thrice for 3 min each, followed by subsequent dehydration and rehydration steps before staining with H&E. Sections were rinsed in water followed by application of Bluing Reagent with further water rinses. Sections were finally immersed in 100% anhydrous alcohol and Clear Rite 3 to improve color stability and cover slips applied accordingly. Slides were examined and images captured on a BioTek Cytation 5 Cell Imaging Multi-Mode Reader.

### Data analysis

Microsphere diameters were analyzed for significant differences by two-tailed student's *t*-test. Cytotoxicity data were analyzed for significant differences by two-way analysis of variance. Pairwise comparisons were evaluated using the Holm-Sidak method. Due to the high variability associated with islets, these data were in violation of the normality and equal variance assumptions. Pairwise comparisons were considered significant at *p* < 0.05 for all comparisons.

## Results

### Core-shell spherification procedure

**FIG. 2.**
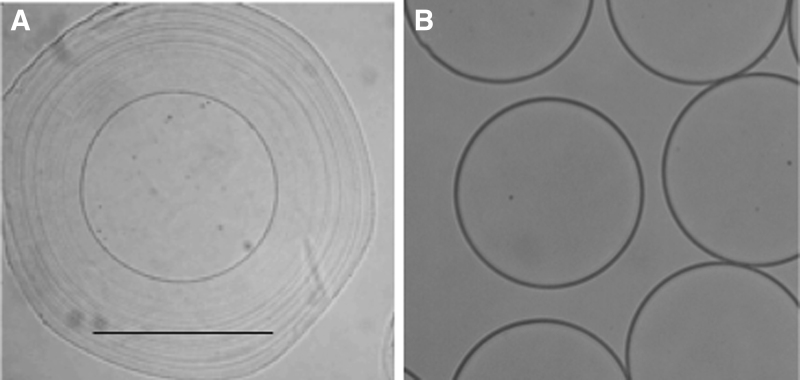
Sample micrographs of core-shell constructs and final microspheres. **(A)** Core-shell constructs have spherical cores and wide, diffuse alginate shells characterized by a concentric ring-like appearance. **(B)** An example of final constructs (PEGDA formulation) after shell removal is shown. Core diameters of constructs produced with the method were generally between 600 and 800 μm. Scale bars = 1 mm. PEGDA, polyethylene glycol diacrylate.

Hydrogel microspheres were fabricated by a novel CSS method summarized in Figure 1. The process starts with the slow-hardening hydrogel material (PEGDA, MeHA, or ThHA) that is entrapped in a shell as it hits the alginate bath (Fig. 1A), providing time for the core hydrogel to harden by either photo or chemical crosslinking (Fig. 1B). An example of the concentric ring-like morphology created by the alginate shell is shown in Figure 2A. The alginate shell is later removed through citrate rinses, resulting in the core construct shown in Figure 2B.

### Physical properties and size distribution of hydrogel microspheres

To fully test the new CSS platform, microbeads were produced from two different starting materials: HA and PEGDA. The HA production was further divided into ThHA and MeHA to evaluate both chemically crosslinked and photo-crosslinked hydrogels, respectively, using the CSS platform. Table 1 provides the physical properties of the hydrogel formulations before and after crosslinking, as well as the average microbead diameter and diameter range. The precursor mass fraction for the gel precursor was 30% for PEGDA, while ThHA and MeHA were fabricated from a 1.2% and 2.5% solution, respectively. The high PEGDA precursor concentration was necessary to achieve a solution viscosity appropriate for forming well-shaped microspheres. Gel formulations and corresponding precursor viscosities are shown in Table 1.

**Table 1.** Microsphere Fabrication Characteristics and Physical Properties

	ThHA microspheres	MeHA microspheres	PEGDA microspheres
Polymer mass fraction in gel precursor	1.2%	2.5% MeHA	18% PEGDA 3.4k
		1% PEGDA 3.4k	12% PEGDA 20k
Precursor viscosity	90 cSt	190 cSt	60 cSt
Crosslinking mechanism	Chemical: thiol-acrylate Michael-type addition	Photo initiated: free radical polymerization	Photo initiated: free radical polymerization
Crosslinking time	35 min	60 s	60 s
Polymer mass fraction in final spheres	3.6%	0.95%	5.8%
Mass swelling ratio “*Q*”^a^	27.7	105.7	17.3
Sphere size range	406–776 μm	1020–1370 μm	424–1146 μm
Average final^b^	637 μm	1156 μm	904 μm
Diameter			
Monodispersity	CV: 11.1%	CV: 6.1%	CV: 15.7%

^a^ Swelling ratio “*Q*” is the ratio of the hydrated equilibrium mass to the dry mass of the gels.

^b^ HA and PEGDA bead diameters were significantly different (*p* < 0.001).

CV, coefficient of variance; HA, hyaluronic acid; PEGDA, polyethylene glycol diacrylate; MeHA, methacrylated HA; ThHA, thiolated HA.

**FIG. 3.**
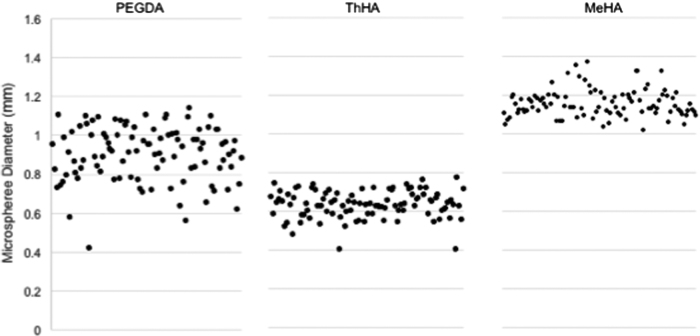
Size distribution. The diameters of a representative sample of 100 microspheres of each material type were measured and displayed in a scatter plot to visualize the size distribution. All microspheres were produced using a 450-μ air jet nozzle system under the same production parameters.

ThHA was the only chemically crosslinked hydrogel and it required significantly more time to crosslink (35 min) compared to the photo-crosslinked gels (MeHA and PEGDA). The average diameter was greatest for the MeHA microspheres, followed by PEGDA and then ThHA microspheres, which were considerably smaller than the other two groups. PEGDA microspheres exhibited the lowest equilibrium swelling ratio, “*Q*,” indicating a more compact overall hydrogel. This is illustrated further in Figure 3, which plots the diameters of 100 representative microspheres composed of ThHA, MeHA, or PEGDA, illustrating good monodispersity of the spheres, especially for the two HA materials.

ThHA microspheres were smaller in size than PEGDA or MeHA, and had a narrower diameter range compared to PEGDA, despite having been fabricated using the same droplet generation equipment and parameters before crosslinking. The average MeHA microsphere diameter was the highest of the three groups. Interestingly, PEGDA spheres containing islets exhibited a slightly larger mean diameter, but improved monodispersity compared to empty PEGDA spheres (938 μm [coefficient of variance (CV): 8.4%] for PEGDA with islets and 904 μm [CV: 15.7%] for empty PEGDA microspheres).

### Diffusion characteristics of microspheres

**FIG. 4.**
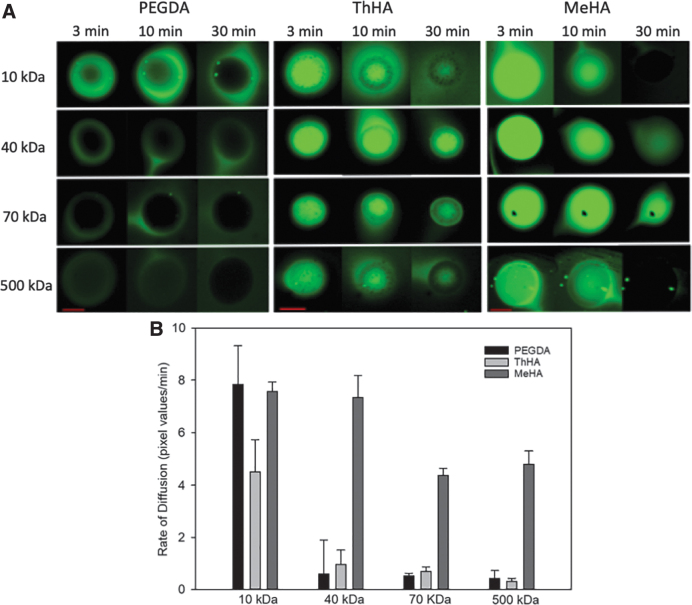
Diffusion of FITC dextrans in hydrogel microspheres. **(A)** Hydrogel microspheres were incubated for 24 h in solutions containing FITC dextrans of increasing molecular weight. After incubation, the FITC-dextran solutions were exchanged with blank DPBS and the microspheres were monitored by confocal microscopy for 150 min to visualize the efflux of the dextran probes. Images of PEGDA, ThHA, and MeHA microspheres captured at the sphere equator are displayed for each condition at 3, 10, and 30-min time points. Scale bars = 500 μm. **(B)** The rate of efflux of the dextran from the core of the spheres was plotted for the first 30 min of the experiment. DPBS, Dulbecco's phosphate-buffered saline; FITC, fluorescein isothiocyanate; MeHA, methacrylated HA; ThHA, thiolated HA.

Figure 4 illustrates the diffusion of fluorescently labeled dextrans into the PEGDA, ThHA and MeHA hydrogel microspheres. Microspheres were incubated overnight in fluorescent dextrans of various molecular weights, rinsed, and immediately examined by confocal microscopy to evaluate the extent of dextran penetration and rate of efflux as a measure of diffusion properties.

The 10 kDa probes were able to penetrate all microspheres. However, fluorescence was weaker in the PEGDA microspheres, specifically near the center of the sphere, suggesting a lower penetration rate of the probe compared to the two HA microspheres, which both displayed strong fluorescence. Furthermore, fluorescence of the 10 kDa probe diminished rapidly in the HA groups and was absent or nearly absent 30 min after rinsing (Fig. 4A). In contrast, localized fluorescence was still observable in the PEGDA group at 30 min, again suggesting a higher diffusion barrier to the 10 kDa probe compared to both HA gels.

Low-level fluorescence from the 40 kDa probe was detected in the periphery of the PEGDA microspheres, but little to no signal was observed at the center, indicating a fairly robust diffusion barrier to the probe. The larger dextrans (70 and 500 kDa) showed little to no fluorescence signal in the PEGDA spheres, suggesting that penetration of these probes into the gel matrix was negligible during the overnight incubation period (Fig. 4A).

Microspheres made of ThHA fluoresced when incubated overnight in each of the probes, including the 500 kDa dextran. Interestingly, the diffusion of the probes out of the microspheres was extremely slow. ThHA microspheres had continued fluorescence even after a 30-min rinse (Fig. 4A). The MeHA microspheres displayed strong fluorescence from all probes evaluated, suggesting minimal diffusion barrier even to large molecules. Furthermore, the relative fluorescence signal in MeHA appeared noticeably higher compared to ThHA for all probes immediately after rinsing, suggesting a higher concentration of the probe, and thus, an even lower diffusion barrier.

The fluorescence intensity at three different locations within the core of each microsphere was measured at 3, 10, 20, and 30 min during the rinsing process and the rate of change calculated. Figure 4B summarizes the rate of fluorescence decline as the dextran diffused out of the microspheres.

Measurements taken at the core of the spheres showed similar rates of fluorescence decline with the 10 kDa dextran. However, at 40 kDa, the rate of decline was much greater for the MeHA microspheres compared to the other two groups. This is due to the fact that there was a higher starting fluorescence intensity level in the MeHA group, and the fluorescence typically dissipated within the 30 min. In contrast, the PEGDA spheres had a low starting fluorescence level for the probes above 10 kDa, and thus, there was little change in fluorescence for the 30 min. Finally, ThHA had more fluorescence, but it was slow to change. These relationships were maintained for the 70 and 500 kDa probes.

### Xenotransplants in diabetic mice

Given the varying physical properties of the microbeads, it was important to identify possible differences in their ability to protect islets and facilitate euglycemia in a xenogeneic transplant model. Thus, canine islets were encapsulated and transplanted into diabetic NOD/SCID mice with a control group that received unencapsulated islets. Current quality assays for islet transplants require >70% viability. Thus, before transplantation, the viability of the encapsulated islets was analyzed.

**FIG. 5.**
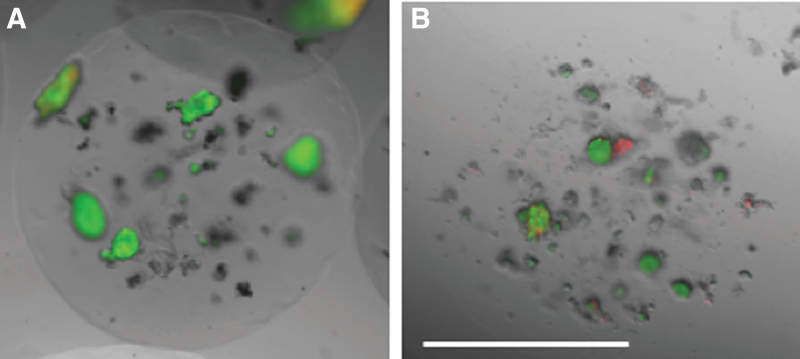
Viability images of microencapsulated islets. Canine islets were encapsulated in PEGDA **(A)** or MeHA **(B)** and stained for live cells with calcein or dead cells using propidium iodide. The majority of cells were viable (*green*) before encapsulation with few dead cells (*red*) noted. Scale bar = 0.5 mm.

Figure 5 provides representative samples of microencapsulated islets stained for live cells using calcein (green) and dead cells using PI (red). Cells within a PEGDA-based microsphere are shown in Figure 5A. The perimeter of the MeHA microbead is difficult to distinguish due to the transparency of these microspheres (Fig. 5B). The average islet viability within 24 h after encapsulation was 84.2% ± 2.2 for the cells encapsulated in PEGDA and 87.6% ± 2.7 for islets in MeHA. There was a range of 6–27 islets/microsphere with no statistical difference between the PEGDA and MeHA groups. Separate encapsulated canine islets were maintained in culture for ∼5 days. At the end of 5 days, the PEGDA encapsulated cells had viability values of 86.5 ± 2.4% and MeHA encapsulated cells were 81.2% ± 2.6 viable, which was not statistically different.

**FIG. 6.**
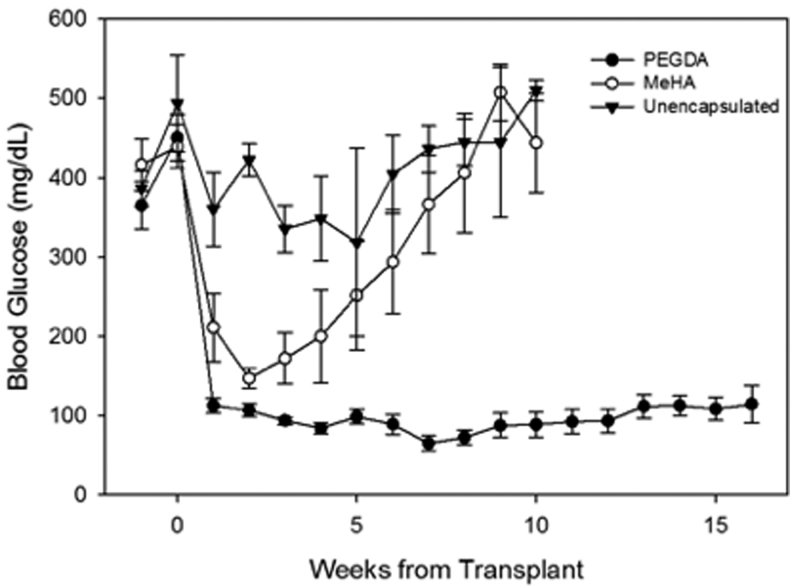
Blood glucose levels of diabetic mice receiving encapsulated islet transplants. Diabetic mice received intraperitoneal transplants of canine islets encapsulated in PEGDA or MeHA microspheres. Blood glucose levels, collected daily for 2 weeks and biweekly for the remainder of the study were calculated as average weekly values. (*N* = 6 PEGDA, 4 MeHA). Week 0 marks the blood glucose reading on the morning of the transplant. Mice in the unencapsulated and MeHA groups were terminated at 10 weeks due to the long-term hyperglycemia.

Figure 6 shows the mean weekly blood glucose levels for each group following the transplant. All mice in both the MeHA and PEGDA groups were normoglycemic by the second week following transplantation. In contrast, all mice that received unencapsulated islets failed to achieve persistent normoglycemia. The animals receiving MeHA microbeads gradually returned to hyperglycemic levels (starting ∼3 weeks post-transplant), despite re-introduction of exogenous insulin therapy. These mice were euthanized at ∼10–11 weeks. Mice receiving PEGDA-encapsulated islets remained normoglycemic throughout the study period with excellent glycemic control without exogenous insulin.

### Explant characteristics

**FIG. 7.**
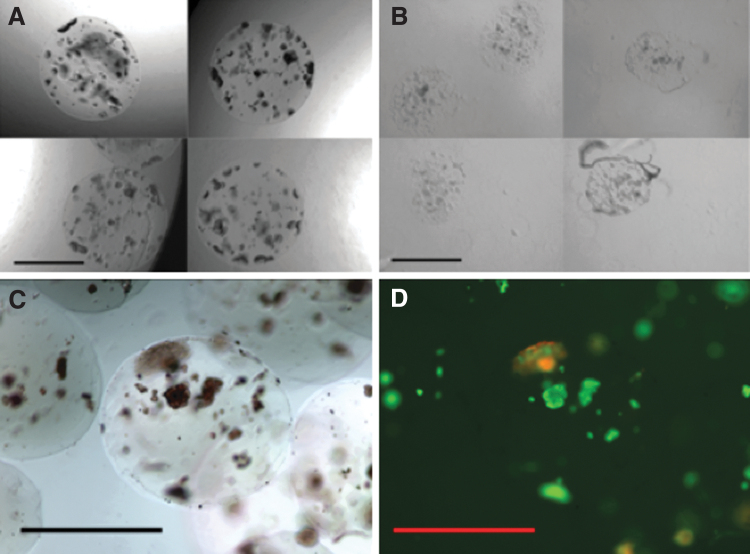
Recovered microspheres after 16 weeks *in vivo*. Microspheres were recovered from the peritoneum of diabetic mice at the 10-week and 16-week time points of the study. **(A)** PEGDA microspheres, retrieved at 10 weeks post-transplant, were spherical and contained dense cellular content within the spheres. **(B)** In contrast, the MeHA microspheres, retrieved at the same time point, were no longer spherical and lacked cellular material. **(C)** An example of a microsphere from the PEGDA group (retrieved 16 weeks post-transplant) was stained with dithizone, indicative of insulin production (*red*). **(D)** Viability was measured by staining the same microsphere with calcein (*green* fluorescence) and propidium iodide (*red* fluorescence). Scale bars = 1 mm.

Upon necropsy, microspheres were recovered from the IP space of the mice. Islet-containing PEGDA microspheres were easily retrieved at 10 and 16 weeks, but the MeHA microspheres were few in number and difficult to identify at 10 weeks. Samples of the retrieved PEGDA microspheres were found randomly dispersed throughout the IP cavity and remained clear and structurally intact. The retrieved PEDGA microspheres showed little to no indication of degradation (i.e., smooth edges, no apparent change in size, and mechanically stable), and the transplanted cells were still contained within the microspheres (Fig. 7A). In contrast, the MeHA microspheres were misshaped and smaller in diameter, indicative of significant hydrogel degradation. The MeHA microspheres did not appear to contain intact islets (Fig. 7B).

At the later time point (16 weeks) the PEDGA microspheres continued to be easily retrievable at necropsy. The islets within the PEGDA microspheres were stained with dithizone, calcein, and PI to confirm the presence of live, functional islets. PEGDA microspheres retrieved at ∼16 weeks following the transplant contained healthy, viable islets identified by deep red dithizone staining (Fig. 7C). The same microspheres was co-labeled green calcein, indicating live cells (Fig. 7D).^32^ Some positive PI staining (red; apoptotic/necrotic cells) was observed, but was generally localized to nonislet (i.e., dithizone negative), often tissue that appeared to be adhered to the surface of the recovered microspheres. Conversely, islets (dithizone-positive cells) were almost completely devoid of PI (dead cell) staining (Fig. 7D).

### Transplant histology

**FIG. 8.**
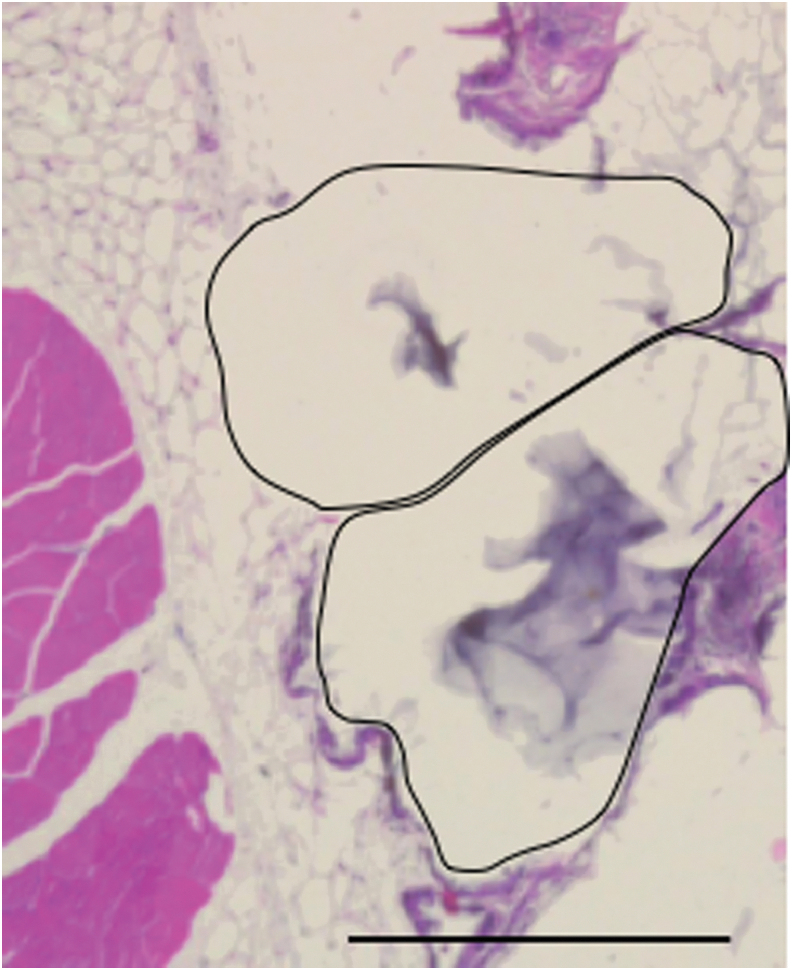
Histology of PEGDA microspheres in diabetic mice. Implanted PEGDA microspheres and surrounding tissues were recovered at 16 weeks. The hematoxylin and eosin histology processing resulted in shrinkage of the microspheres and the region that had contained two microspheres is shown outlined in the image (*black outlines*). The *gray* material in the center of each region is the remaining hydrogel. There is an absence of inflammatory cells and fibroblasts around the microspheres' location. Scale bar = 500 μm.

PEGDA microspheres were occasionally found attached to tissues such as the liver and abdominal wall, but were generally unattached and free floating in the cavity. MeHA microspheres were not found attached to surrounding tissues. Histology of the graft site identified the location of the PEGDA microspheres. While the method of histology processing destroyed the integrity of the microspheres, the attachment sites were free of obvious fibrotic tissue. Figure 8 illustrates healthy adipocytes at the intersection with two microspheres, with the location of the microspheres denoted by the black lines. The bluish gray-colored film in the center of each microsphere location is the remaining hydrogel following processing. There was a general lack of fibrosis at the microsphere-omentum interface. Only the lower region of one microsphere showed minimal signs of a low-grade local inflammation.

Evaluation of tissue samples retrieved at 10 or 16 weeks post-transplant with PEGDA encapsulation showed no differences at the tissue/microsphere interface.

To ensure that the normalized blood glucose values in the PEGDA-treated mice were due to the graft transplant, rather than endogenous islets remaining within the pancreas after the STZ treatment, histological sections of the pancreas were examined for the presence of islets. Only one islet-like structure was identified in six pancreatic sections per mouse (results not shown).

## Discussion

We developed and evaluated a new method for fabricating hydrogel microspheres for cell encapsulation and delivery. Current methods for cell microencapsulation are predominantly based on alginate spheres because of the quick gelation rate.^33,34^ Other hydrogels utilized for microencapsulation involve oil-emulsion techniques,^13,35^ which can have issues with toxicity to cells. Alternatively, our method, termed CSS, could be used for a wide variety of hydrogel materials and is compatible with standard, commercially available and GMP-ready equipment, and is minimally cytotoxic to islets.

The CSS method of microsphere production is based on the same basic fabrication methods as traditional alginate beads, which start with cells mixed with an alginate precursor solution. The mixture is then dropped by syringe or microencapsulator, such as the Buchi used in this study, into a bath of CaCl_2_ (the crosslinker for alginate). Upon contact with the calcium, the alginate quickly hardens into the gel form. As described in the introduction, there are a multitude of variations on this procedure, with CSS adding a new option to the field.

The microspheres produced by CSS using different materials were significantly different in size, with MeHA beads nearly twice the diameter of ThHA beads, corresponding to a roughly eight-fold difference in total volume per sphere, despite being fabricated using the same 400-μ nozzle system. The difference in size between the groups was most likely a result of differences in postfabrication volumetric swelling dynamics. Hydrogel swelling is governed by a number of variables, but is strongly correlated to initial (i.e., precrosslinked) concentration of polymer and the density of crosslinks within the gel after fabrication.^30,31^

More specifically, solvent molecules (e.g., water) adsorbed to the gel polymer matrix exert outward pressure, causing the gel to expand (swell), whereas crosslinks in the gel matrix resist outward expansion. For example, a hydrogel with high initial polymer concentration and a low crosslink density would be expected to expand significantly in volume after fabrication.^36^ Conversely, gels with a high ratio of crosslinks to polymer concentration tend to resist expansion after fabrication. In our study, the smaller final size of the ThHA spheres was likely a result of a low initial polymer fraction (1.2%) coupled with relatively high crosslink density.

Diffusion properties were considerably different between the microsphere groups, which were best visualized in the example images. Analysis of the images for absolute levels and rates of dextran diffusion should be taken with caution. First, the fluorescence intensity is directly related to the thickness of the hydrogel, even when imaged with confocal microscopy. Thus, part of the reason for the higher fluorescence level in the MeHA microspheres could be due to the fact that the spheres swelled more and thus were thicker (greater diameter) (Fig. 4). The rate calculations provided are best utilized when comparing within hydrogel. For example, Figure 4B illustrates that the PEGDA microspheres had a higher rate of diffusion for the 10 kDa dextran compared to any of the size probes, whereas MeHA had similar diffusion rates for both the 70 and 500 kDa probes.

However, one can see from the images that ThHA and MeHA microspheres were highly permeable to dextran probes up to 500 kDa in size, with MeHA microspheres appearing to have the highest diffusivity of all three groups. Conversely, PEGDA spheres appeared to strongly limit diffusion of dextrans of MW 40 kDa and larger. Although a wide array of factors can affect diffusivity of a gel, the swelling ratio “*Q*” (the ratio of the equilibrium hydrated mass to the dry mass of the gel) is strongly correlated to the permeability of the hydrogel.^37,38^ The PEGDA, ThHA, and MeHA microspheres had *Q* values of 17.3, 27.7, and 105.7, respectively. Thus, the diffusion behavior of the dextran probes observed in the microspheres is consistent with their corresponding *Q* values.

We utilized a canine to mouse islet xenograft model to evaluate the *in vivo* functional capacity of cells encapsulated by CSS in both MeHA and PEGDA. An immune-deficient NOD/SCID mouse was used to eliminate potentially confounding immunological variables, enabling evaluation of the effects of different encapsulation materials on transplant function. Interestingly, while both encapsulated islet groups achieved normoglycemia after IP injection of the microspheres, unencapsulated canine islets at the same dose and transplant site did not achieve normoglycemia. Because an immune response was not present in this model, this result suggests that the hydrogel matrices provide a physically supportive environment that facilitates better cell function and survival, particularly in the context of transplantation by injection into the IP cavity.

The differences in the *in vivo* stability of the different microsphere formulations are clearly apparent in the duration of normal blood glucose levels in the three groups. The observation of degradation of the MeHA microspheres over 10 weeks with concurrent reversing to hyperglycemia illustrates the short *in vivo* duration of the MeHA spheres.

In contrast, the PEGDA-islet microspheres, which were more structurally robust, led to long-term control of blood glucose levels compared to MeHA transplants. These results indicate that the physical properties of the microsphere matrices are important to facilitate proper function of transplanted islets regardless of an immune response. While these data indicate that these MeHA microspheres were not suitable for islet transplantation (where an ideal graft would persist indefinitely), this material could have utility in cell transplantation applications that are not intended to be permanent, such as wound healing or localized stem cell delivery.

## Conclusion

In conclusion, the novel method presented herein for producing hydrogel microspheres is a new tool for cell transplantation and tissue engineering research. CSS could be applied to a wide variety of hydrogel materials, thereby enabling broad control of microsphere properties for application-specific purposes. In this study, microspheres were produced with markedly different size, structural, and mass transport properties with minimal modifications to the fabrication protocol.

Islets encapsulated in PEGDA by CSS successfully reversed diabetes in a mouse xenograft model, and intact microspheres containing healthy and viable islets were recovered from the mice after 16 weeks. The microspheres evoked no negative effect *in vivo* and appeared to be well tolerated. Together these data demonstrate the value and versatility of the CSS method for delivery and support of therapeutic cells, including transplantation of islets for diabetes.
